# Pulmonary Acantholytic Squamous Cell Carcinoma Mimicking Lepidic Pattern Adenocarcinoma

**DOI:** 10.5146/tjpath.2019.01486

**Published:** 2021-01-15

**Authors:** Rukiye Yılmaz, Recep Bedir

**Affiliations:** Recep Tayyip Erdoğan University School of Medicine, Department of Pathology, Rize, Turkey

**Figure 1:**
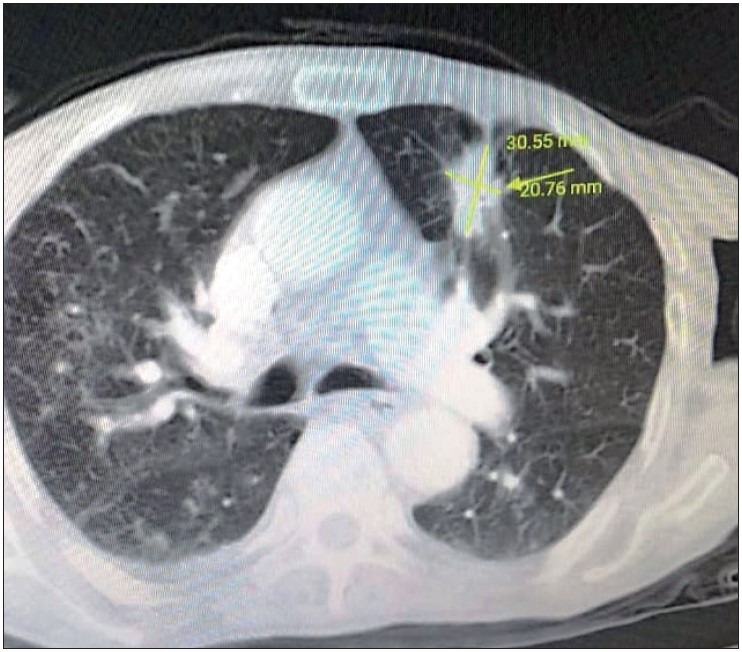
CT revealed a mass of 3x2 cm on the superior lobe of the left lung (yellow arrow).

Dear Editor,

An 84-year-old male patient with a history of smoking and recent pneumonia symptoms of fever, difficulty in breathing, and weakness presented to the chest disease clinic. The patient had a left-sided suspicious lung mass on his chest X-Ray (CXR). Written informed consent was obtained from the patient. Chest computed tomography (CT) revealed a mass of 3x2 cm on the superior lobe of the left lung (Figure 1). CT-guided transthoracic tru-cut biopsy of the chest was performed.

**Figure 2:**
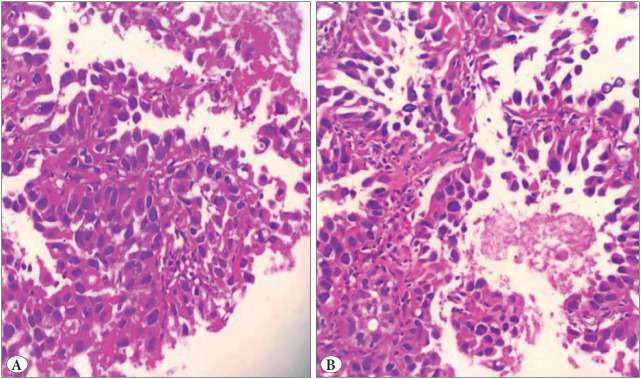
**A-B)** Atypical epithelial cells with large hyperchromatic and pleomorphic nucleus, showing acantholytic changes and forming gland-like structures (H&E; x400).

**Figure 3:**
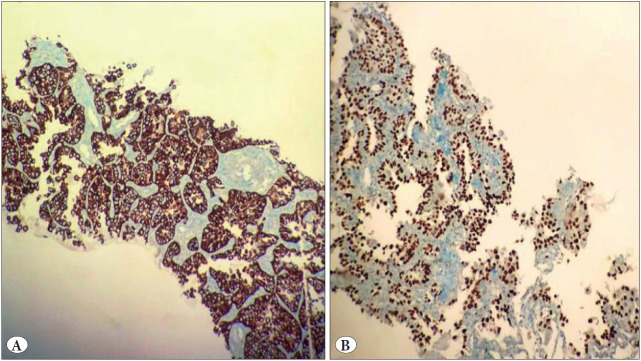
Neoplastic cells showed diffuse positivity for **A)** CK7 (IHC; x200) and **B)** p40 (IHC; x200)

Microscopic examination revealed an infiltrative tumor consisting of atypical epithelial cells with a large hyper-chromatic nucleus and showing prominent pleomorphism and acantholytic changes with gland-like structures in the lung parenchyma (Figure 2). Immunohistochemical evaluation revealed diffuse positive staining with p40 and cytokeratin (CK) 7 (Figure 3) while TTF-1, GATA-3 and CK20 were negative in the neoplastic cells. GATA-3 and CK20 immunohistochemical staining was performed as the tumor contained some urothelial carcinoma-like areas. Urothelial carcinoma metastasis was eliminated from the differential diagnosis as these markers were negative. A diagnosis of adenocarcinoma was eliminated and a diagnosis of squamous cell carcinoma was supported as a result of the negative staining with TTF-1 and diffuse strong positive staining with p40. With these findings, the case was reported as squamous cell carcinoma (acantholytic/adenoid-like variant). An irregular bordered pathological lesion (SUVmax:10.4), with an axial long border of about 3 cm, and creating traction on the mediastinal and costal pleura, was observed in the anterior segment of the superior lobe of the left lung. There was no evidence to suggest non-pulmonary SCC in other organs in the clinical or radiological signs. The patient was considered to be inoperable because of the distant metastases and chemotherapy treatment was planned.

SCC of the lung is a histological type of non-small cell lung carcinoma. It is one of the most prevalent lung cancers and originates from the bronchi. SCC infrequently presents with acantholysis, which is characterized by loosening of the cell-to-cell connection. Acantholytic SCC is a rare variant, and arises most commonly in the skin. The acantholytic variant of SCC is rarely seen in the skin and also has an extremely rare presentation in the lung with only a few cases reported in the literature. Since the artefactual clefts of the tumoral cells could resemble acantholysis and glandular lumens or a vascular structure, acantholytic SCC is also called as adenoid / pseudovascular / pseudovascular adenoid SCC (1–3). We aimed to report our case as it is very rare in the literature.

Acantholytic SCC comprises 2-4% of all cutaneous SCCs. Many skin pathology textbooks histologically characterize SCC as adenoid (pseudoglandular) or pseudoacinar nests with central acantholysis and cohesive peripheral tumor cells. The skin is the most frequent site of acantholytic tumors, with common skin pathology references. Pulmonary acantholytic SCC results in an aggressive clinical course, with marked lymphatic metastases (4,5). p40 has high immunohistochemical sensitivity and specificity to distinguish lung adenocarcinoma and SCC and appears to be an excellent marker for SCC. p40 immunostaining should be performed routinely for the diagnosis of pulmonary SCC (6). The positive expression rates of TTF-1 and NapsinA are higher in lung adenocarcinoma, and TTF-1 is highly specific and sensitive in the diagnosis of adenocarcinoma. NapsinA may be used to distinguish ADC and SCC (7).

In conclusion, the acantholytic/adenoid-like variant of SCC should be kept in mind during evaluation of lung biopsies, and careful microscopic examination and immunohistochemical evaluation should be performed since the tumor can mimic the lepidic pattern and other adenocarcinomas. The immunoexpression profile of SCC can help in making the correct diagnosis.
